# Dimethyl 2-(2-quinolylmeth­yl)malonate

**DOI:** 10.1107/S1600536810013930

**Published:** 2010-05-19

**Authors:** Shou-Xin Liu, Zhao-Hui Zhang, Yu-Feng Yang, Xiao-Li Zhen, Jian-Rong Han

**Affiliations:** a College of Chemical & Pharmaceutical Engineering, Hebei University of Science & Technology, Shijiazhuang 050018, People’s Republic of China; b College of Sciences, Hebei University of Science & Technology, Shijiazhuang 050018, People’s Republic of China

## Abstract

In the title compound, C_15_H_15_NO_4_, the quinoline ring system and one of the malonate side chains are essentially coplanar (r.m.s. deviation = 0.0297 Å). The two malonate C—C(=O)—O—CH_3_ side chains are oriented at right angles [89.68 (8)°] with respect to each other. The crystal packing is stabilized by weak non-classical inter­molecular C—H⋯O hydrogen bonds, which link the mol­ecules into dimers about inversion centers.

## Related literature

For general background to the synthesis of halomalonates, see: Okimoto & Takahashi (2002 ▶). 
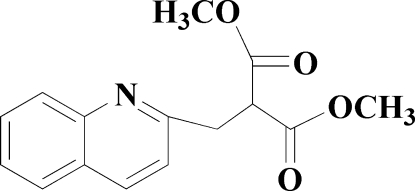


## Experimental

### 

#### Crystal data

C_15_H_15_NO_4_
*M* *_r_* = 273.28
Monoclinic, 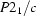

*a* = 10.626 (2) Å
*b* = 16.198 (3) Å
*c* = 8.1859 (16) Å
β = 107.92 (3)°
*V* = 1340.6 (5) Å^3^
*Z* = 4
Mo *K*α radiation
μ = 0.10 mm^−1^
*T* = 113 K
0.20 × 0.18 × 0.12 mm

#### Data collection

Rigaku Saturn CCD area-detector diffractometer
Absorption correction: multi-scan (*CrystalClear*; Rigaku/MSC, 2005 ▶) *T*_min_ = 0.981, *T*_max_ = 0.988
8841 measured reflections
2355 independent reflections
2094 reflections with *I* > 2σ(*I*)
*R*_int_ = 0.033

#### Refinement

*R*[*F* ^2^ > 2σ(*F* ^2^)] = 0.035
*wR*(*F* ^2^) = 0.095
*S* = 1.07
2355 reflections
184 parameters
H-atom parameters constrained
Δρ_max_ = 0.23 e Å^−3^
Δρ_min_ = −0.17 e Å^−3^

### 

Data collection: *CrystalClear* (Rigaku/MSC, 2005 ▶); cell refinement: *CrystalClear*; data reduction: *CrystalClear*; program(s) used to solve structure: *SHELXS97* (Sheldrick, 2008 ▶); program(s) used to refine structure: *SHELXL97* (Sheldrick, 2008 ▶); molecular graphics: *SHELXTL* (Sheldrick, 2008 ▶); software used to prepare material for publication: *SHELXTL*.

## Supplementary Material

Crystal structure: contains datablocks I, global. DOI: 10.1107/S1600536810013930/pv2271sup1.cif

Structure factors: contains datablocks I. DOI: 10.1107/S1600536810013930/pv2271Isup2.hkl

Additional supplementary materials:  crystallographic information; 3D view; checkCIF report

## Figures and Tables

**Table 1:** Hydrogen-bond geometry (Å, °)

*D*—H⋯*A*	*D*—H	H⋯*A*	*D*⋯*A*	*D*—H⋯*A*
C11—H11⋯O1^i^	0.98	2.49	3.410 (2)	157

Symmetry code: (i) 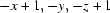.

**Fig. 1.**
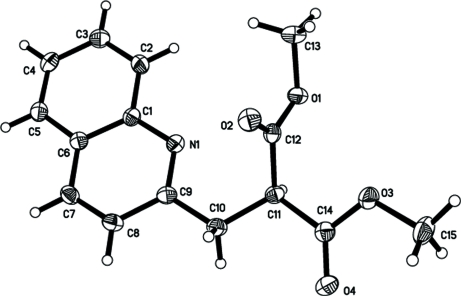
Ellipsoid plot.

**Fig. 2.**
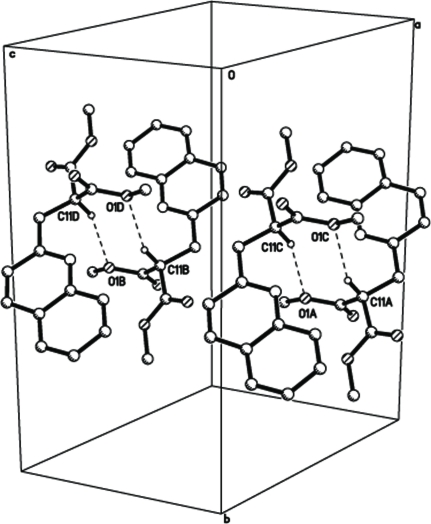
Packing diagram.

## supplementary crystallographic information

### Comment

Substituted malonate is a very important organic intermediate. It is electrooxidized in methanol in the presence of halogen ions to afford the corresponding halomalonates (Okimoto & Takahashi, 2002). We have synthesized the title compound, (I), in our laboratory and report in this article its synthesis and crystal structure.

The molecular structure of (I) is presented in Fig. 1. In (I), the quinoline ring and the side chain atoms C10/C11/C14 are essentially planar [r.m.s. deviation, 0.0297 Å]. The two malonate side chains comprising C/C/O/C atoms (C11/C12/O1/C13 and C11/C14/O3/C15) are oriented at right angles (89.68 (8)°) with respect to each other. The crystal packing is stabilized by weak non-classical intermolecular C—H···O hydrogen bonds which link the molecules into dimers about inversion centers.

### Experimental

An anhydrous methanol solution (130 ml) of 2-brommethyl quinoline was added to an anhydrous methanol solution (180 ml) of sodium methoxide (5.4 g, 0.1 mol) and dimethyl malonate (26.4 g, 0.2 mol). The mixture was refluxed for 4 h and the product was isolated with silica gel column. The solvent was removed and a little petroleum ether was added to the resultant to give pale-yellow precipitates which were isolated, recrystallized from n-hexane, and dried under vacuum to give the title compound (60% yield). Colorless single crystals of the title compound suitable for X-ray analysis were obtained by slow evaporation of an n-hexane solution.

### Refinement

The H atoms were included in calculated positions (C—H = 0.93–0.98 Å) and refined as riding with U_iso_(H) = 1.2U_eq_(C) or 1.5U_eq_(methyl C).

### Crystal data

**Table:**

C_15_H_15_NO_4_	*F*(000) = 576
*M**_r_* = 273.28	*D*_x_ = 1.354 Mg m^−^^3^
Monoclinic, *P*2_1_/*c*	Mo *K*α radiation, λ = 0.71073 Å
Hall symbol: -P 2ybc	Cell parameters from 4229 reflections
*a* = 10.626 (2) Å	θ = 2.0–27.9°
*b* = 16.198 (3) Å	µ = 0.10 mm^−^^1^
*c* = 8.1859 (16) Å	*T* = 113 K
β = 107.92 (3)°	Prism, colourless
*V* = 1340.6 (5) Å^3^	0.20 × 0.18 × 0.12 mm
*Z* = 4	

### Data collection

**Table:**

Rigaku Saturn CCD area-detector diffractometer	2355 independent reflections
Radiation source: rotating anode	2094 reflections with *I* > 2σ(*I*)
confocal	*R*_int_ = 0.033
Detector resolution: 7.31 pixels mm^-1^	θ_max_ = 25.0°, θ_min_ = 2.4°
ω and φ scans	*h* = −12→7
Absorption correction: multi-scan (*CrystalClear*; Rigaku/MSC, 2005)	*k* = −19→18
*T*_min_ = 0.981, *T*_max_ = 0.988	*l* = −9→9
8841 measured reflections	

### Refinement

**Table:**

Refinement on *F*^2^	Secondary atom site location: difference Fourier map
Least-squares matrix: full	Hydrogen site location: inferred from neighbouring sites
*R*[*F*^2^ > 2σ(*F*^2^)] = 0.035	H-atom parameters constrained
*wR*(*F*^2^) = 0.095	*w* = 1/[σ^2^(*F*_o_^2^) + (0.0544*P*)^2^ + 0.2353*P*] where *P* = (*F*_o_^2^ + 2*F*_c_^2^)/3
*S* = 1.07	(Δ/σ)_max_ = 0.003
2355 reflections	Δρ_max_ = 0.23 e Å^−^^3^
184 parameters	Δρ_min_ = −0.17 e Å^−^^3^
0 restraints	Extinction correction: *SHELXL97* (Sheldrick, 2008), Fc^*^=kFc[1+0.001xFc^2^λ^3^/sin(2θ)]^-1/4^
Primary atom site location: structure-invariant direct methods	Extinction coefficient: 0.133 (8)

### Special details

**Table:**

Geometry. All esds (except the esd in the dihedral angle between two l.s. planes) are estimated using the full covariance matrix. The cell esds are taken into account individually in the estimation of esds in distances, angles and torsion angles; correlations between esds in cell parameters are only used when they are defined by crystal symmetry. An approximate (isotropic) treatment of cell esds is used for estimating esds involving l.s. planes.
Refinement. Refinement of *F*^2^ against ALL reflections. The weighted *R*-factor wR and goodness of fit *S* are based on *F*^2^, conventional *R*-factors *R* are based on *F*, with *F* set to zero for negative *F*^2^. The threshold expression of *F*^2^ > σ(*F*^2^) is used only for calculating *R*-factors(gt) etc. and is not relevant to the choice of reflections for refinement. *R*-factors based on *F*^2^ are statistically about twice as large as those based on *F*, and *R*- factors based on ALL data will be even larger.

### Fractional atomic coordinates and isotropic or equivalent isotropic displacement parameters (Å^2^)

**Table:**

	*x*	*y*	*z*	*U*_iso_*/*U*_eq_	
O1	0.42646 (9)	0.11002 (5)	0.37324 (10)	0.0208 (2)	
O2	0.28197 (9)	0.17212 (5)	0.48439 (11)	0.0238 (3)	
O3	0.57807 (9)	0.20028 (5)	0.71040 (11)	0.0259 (3)	
O4	0.58734 (10)	0.12269 (6)	0.94065 (12)	0.0315 (3)	
N1	0.24000 (10)	−0.02029 (6)	0.47394 (13)	0.0193 (3)	
C1	0.14301 (12)	−0.06967 (7)	0.37093 (16)	0.0186 (3)	
C2	0.13159 (14)	−0.07574 (8)	0.19515 (17)	0.0249 (3)	
H2	0.1898	−0.0466	0.1519	0.030*	
C3	0.03559 (14)	−0.12414 (8)	0.08742 (17)	0.0275 (3)	
H3	0.0287	−0.1271	−0.0285	0.033*	
C4	−0.05277 (13)	−0.16937 (8)	0.15063 (17)	0.0240 (3)	
H4	−0.1164	−0.2029	0.0771	0.029*	
C5	−0.04480 (13)	−0.16388 (8)	0.32021 (17)	0.0217 (3)	
H5	−0.1036	−0.1937	0.3612	0.026*	
C6	0.05144 (13)	−0.11361 (7)	0.43356 (16)	0.0186 (3)	
C7	0.06142 (13)	−0.10242 (8)	0.60895 (16)	0.0220 (3)	
H7	0.0030	−0.1293	0.6554	0.026*	
C8	0.15648 (13)	−0.05239 (8)	0.70838 (16)	0.0224 (3)	
H8	0.1631	−0.0444	0.8232	0.027*	
C9	0.24603 (12)	−0.01216 (7)	0.63656 (15)	0.0189 (3)	
C10	0.35008 (13)	0.04359 (8)	0.74929 (15)	0.0210 (3)	
H10A	0.4004	0.0127	0.8496	0.025*	
H10B	0.3065	0.0886	0.7886	0.025*	
C11	0.44620 (12)	0.07996 (7)	0.66261 (15)	0.0183 (3)	
H11	0.4954	0.0344	0.6324	0.022*	
C12	0.37365 (12)	0.12590 (7)	0.49881 (15)	0.0182 (3)	
C13	0.35706 (15)	0.14582 (9)	0.20836 (16)	0.0306 (3)	
H13A	0.2709	0.1213	0.1651	0.046*	
H13B	0.4060	0.1357	0.1295	0.046*	
H13C	0.3481	0.2042	0.2211	0.046*	
C14	0.54437 (12)	0.13576 (8)	0.78861 (16)	0.0203 (3)	
C15	0.67016 (15)	0.25858 (9)	0.81881 (19)	0.0318 (4)	
H15A	0.6337	0.2801	0.9038	0.048*	
H15B	0.6855	0.3030	0.7498	0.048*	
H15C	0.7522	0.2312	0.8747	0.048*	

### Atomic displacement parameters (Å^2^)

**Table:**

	*U*^11^	*U*^22^	*U*^33^	*U*^12^	*U*^13^	*U*^23^
O1	0.0226 (5)	0.0238 (5)	0.0158 (5)	0.0004 (4)	0.0055 (4)	0.0020 (3)
O2	0.0218 (5)	0.0216 (5)	0.0273 (5)	0.0050 (4)	0.0067 (4)	0.0024 (4)
O3	0.0279 (5)	0.0222 (5)	0.0243 (5)	−0.0075 (4)	0.0032 (4)	−0.0002 (4)
O4	0.0320 (6)	0.0367 (6)	0.0204 (5)	−0.0099 (4)	0.0004 (4)	0.0007 (4)
N1	0.0180 (6)	0.0198 (6)	0.0194 (5)	0.0002 (4)	0.0048 (4)	0.0000 (4)
C1	0.0166 (7)	0.0180 (6)	0.0205 (6)	0.0025 (5)	0.0046 (5)	0.0006 (5)
C2	0.0233 (7)	0.0314 (7)	0.0216 (7)	−0.0043 (6)	0.0095 (6)	−0.0014 (5)
C3	0.0283 (8)	0.0353 (8)	0.0188 (7)	−0.0035 (6)	0.0070 (6)	−0.0043 (6)
C4	0.0198 (7)	0.0245 (7)	0.0247 (7)	−0.0015 (5)	0.0021 (6)	−0.0039 (5)
C5	0.0190 (7)	0.0194 (6)	0.0262 (7)	0.0003 (5)	0.0062 (6)	0.0026 (5)
C6	0.0179 (7)	0.0156 (6)	0.0218 (6)	0.0039 (5)	0.0054 (5)	0.0032 (5)
C7	0.0240 (7)	0.0212 (7)	0.0224 (7)	−0.0013 (5)	0.0094 (6)	0.0043 (5)
C8	0.0272 (7)	0.0225 (6)	0.0176 (6)	0.0000 (5)	0.0072 (6)	0.0024 (5)
C9	0.0207 (7)	0.0166 (6)	0.0184 (6)	0.0035 (5)	0.0045 (5)	0.0022 (5)
C10	0.0243 (7)	0.0212 (6)	0.0171 (6)	−0.0012 (5)	0.0059 (5)	0.0012 (5)
C11	0.0193 (7)	0.0174 (6)	0.0173 (6)	0.0010 (5)	0.0044 (5)	−0.0003 (5)
C12	0.0189 (7)	0.0155 (6)	0.0197 (6)	−0.0034 (5)	0.0052 (5)	−0.0015 (5)
C13	0.0328 (8)	0.0384 (8)	0.0182 (7)	−0.0002 (6)	0.0044 (6)	0.0070 (6)
C14	0.0180 (7)	0.0215 (7)	0.0212 (7)	0.0028 (5)	0.0059 (5)	0.0002 (5)
C15	0.0280 (8)	0.0252 (7)	0.0368 (8)	−0.0083 (6)	0.0019 (7)	−0.0041 (6)

### Geometric parameters (Å, °)

**Table:**

O1—C12	1.3389 (15)	C6—C7	1.4184 (18)
O1—C13	1.4454 (16)	C7—C8	1.3542 (19)
O2—C12	1.2054 (15)	C7—H7	0.9300
O3—C14	1.3309 (15)	C8—C9	1.4212 (18)
O3—C15	1.4500 (16)	C8—H8	0.9300
O4—C14	1.2052 (16)	C9—C10	1.5039 (18)
N1—C9	1.3195 (16)	C10—C11	1.5296 (17)
N1—C1	1.3705 (17)	C10—H10A	0.9700
C1—C2	1.4098 (18)	C10—H10B	0.9700
C1—C6	1.4222 (18)	C11—C14	1.5189 (18)
C2—C3	1.3710 (19)	C11—C12	1.5197 (17)
C2—H2	0.9300	C11—H11	0.9800
C3—C4	1.4089 (19)	C13—H13A	0.9600
C3—H3	0.9300	C13—H13B	0.9600
C4—C5	1.3673 (18)	C13—H13C	0.9600
C4—H4	0.9300	C15—H15A	0.9600
C5—C6	1.4093 (19)	C15—H15B	0.9600
C5—H5	0.9300	C15—H15C	0.9600
			
C12—O1—C13	115.21 (10)	C9—C10—C11	114.61 (10)
C14—O3—C15	116.60 (10)	C9—C10—H10A	108.6
C9—N1—C1	118.22 (11)	C11—C10—H10A	108.6
N1—C1—C2	118.74 (11)	C9—C10—H10B	108.6
N1—C1—C6	122.59 (11)	C11—C10—H10B	108.6
C2—C1—C6	118.65 (12)	H10A—C10—H10B	107.6
C3—C2—C1	120.65 (12)	C14—C11—C12	111.33 (10)
C3—C2—H2	119.7	C14—C11—C10	109.35 (10)
C1—C2—H2	119.7	C12—C11—C10	111.59 (10)
C2—C3—C4	120.61 (12)	C14—C11—H11	108.1
C2—C3—H3	119.7	C12—C11—H11	108.1
C4—C3—H3	119.7	C10—C11—H11	108.1
C5—C4—C3	119.93 (12)	O2—C12—O1	124.13 (11)
C5—C4—H4	120.0	O2—C12—C11	124.46 (11)
C3—C4—H4	120.0	O1—C12—C11	111.39 (10)
C4—C5—C6	120.78 (12)	O1—C13—H13A	109.5
C4—C5—H5	119.6	O1—C13—H13B	109.5
C6—C5—H5	119.6	H13A—C13—H13B	109.5
C5—C6—C7	123.60 (12)	O1—C13—H13C	109.5
C5—C6—C1	119.35 (11)	H13A—C13—H13C	109.5
C7—C6—C1	117.04 (12)	H13B—C13—H13C	109.5
C8—C7—C6	119.63 (11)	O4—C14—O3	124.21 (12)
C8—C7—H7	120.2	O4—C14—C11	124.03 (12)
C6—C7—H7	120.2	O3—C14—C11	111.76 (10)
C7—C8—C9	119.76 (11)	O3—C15—H15A	109.5
C7—C8—H8	120.1	O3—C15—H15B	109.5
C9—C8—H8	120.1	H15A—C15—H15B	109.5
N1—C9—C8	122.73 (12)	O3—C15—H15C	109.5
N1—C9—C10	118.51 (11)	H15A—C15—H15C	109.5
C8—C9—C10	118.74 (11)	H15B—C15—H15C	109.5
			
C9—N1—C1—C2	177.02 (11)	C7—C8—C9—N1	1.02 (19)
C9—N1—C1—C6	−1.52 (17)	C7—C8—C9—C10	179.55 (11)
N1—C1—C2—C3	−179.71 (12)	N1—C9—C10—C11	−4.71 (16)
C6—C1—C2—C3	−1.11 (19)	C8—C9—C10—C11	176.70 (11)
C1—C2—C3—C4	−0.6 (2)	C9—C10—C11—C14	179.17 (10)
C2—C3—C4—C5	1.3 (2)	C9—C10—C11—C12	55.55 (14)
C3—C4—C5—C6	−0.27 (19)	C13—O1—C12—O2	−6.25 (17)
C4—C5—C6—C7	177.20 (12)	C13—O1—C12—C11	175.19 (10)
C4—C5—C6—C1	−1.43 (18)	C14—C11—C12—O2	−79.24 (15)
N1—C1—C6—C5	−179.36 (11)	C10—C11—C12—O2	43.25 (16)
C2—C1—C6—C5	2.10 (17)	C14—C11—C12—O1	99.32 (11)
N1—C1—C6—C7	1.92 (17)	C10—C11—C12—O1	−138.19 (11)
C2—C1—C6—C7	−176.62 (11)	C15—O3—C14—O4	−1.50 (18)
C5—C6—C7—C8	−179.49 (12)	C15—O3—C14—C11	179.13 (10)
C1—C6—C7—C8	−0.82 (18)	C12—C11—C14—O4	159.19 (13)
C6—C7—C8—C9	−0.55 (19)	C10—C11—C14—O4	35.42 (17)
C1—N1—C9—C8	0.03 (17)	C12—C11—C14—O3	−21.44 (14)
C1—N1—C9—C10	−178.51 (10)	C10—C11—C14—O3	−145.21 (10)

### Hydrogen-bond geometry (Å, °)

**Table:**

*D*—H···*A*	*D*—H	H···*A*	*D*···*A*	*D*—H···*A*
C11—H11···O1^i^	0.98	2.49	3.410 (2)	157

Symmetry codes: (i) −*x*+1, −*y*, −*z*+1.
